# A proton conductor electrolyte based on molten CsH_5_(PO_4_)_2_ for intermediate-temperature fuel cells

**DOI:** 10.1039/c7ra12803g

**Published:** 2018-01-30

**Authors:** Xiaojing Chen, Yichong Zhang, Paulo Ribeiorinha, Haibin Li, Xiangyang Kong, Marta Boaventura

**Affiliations:** School of Materials Science and Engineering, Shanghai Jiao Tong University 800 Dong Chuan Road Shanghai 200240 China; State Key Laboratory of Ocean Engineering, Shanghai Jiao Tong University 800 Dongchuan Road Shanghai 200240 China haibinli@sjtu.edu.cn; Collaborative Innovation Center for Advanced Ship and Deep-Sea Exploration, Shanghai Jiao Tong University 800 Dongchuan Road Shanghai 200240 China; LEPABE, Faculdade de Engenharia, Universidade do Porto Rua Dr. Roberto Frias 4200-465 Porto Portugal marta.boaventura@fe.up.pt

## Abstract

Molten carbonate fuel cells have been commercialized as a mature technology. Due to the liquid electrolyte in molten carbonate fuel cells, gas seal and low contact resistance are easier to achieve than in other fuel cells. Herein, we report an investigation of the viability of a molten oxoacid salt as a novel type of fuel cell electrolyte. In comparison with molten carbonate electrolytes for MCFCs that operate at 500–700 °C, for which a ceramic support matrix is required, the molten proton conductor electrolyte has a lower working temperature range of 150–250 °C. The present study has shown that an electrolyte membrane, in which molten CsH_5_(PO_4_)_2_ is held in a matrix made of PBI polymer and SiO_2_ powder, has excellent thermal stability, good mechanical properties, and high proton conductivity. In addition, a molten proton conductor fuel cell equipped with such an electrolyte membrane operating at 200 °C showed an open-circuit voltage of 1.08 V, and a stable output voltage during continuous measurement for 150 h at a constant output current density of 100 mA cm^−2^.

## Abstract 2

An proton conductor electrolyte membrane, in which molten CsH_5_(PO_4_)_2_ is held in a matrix made of PBI polymer and SiO_2_ powder, is prepared for intermediate-temperature fuel cells.

## Graphical abstract

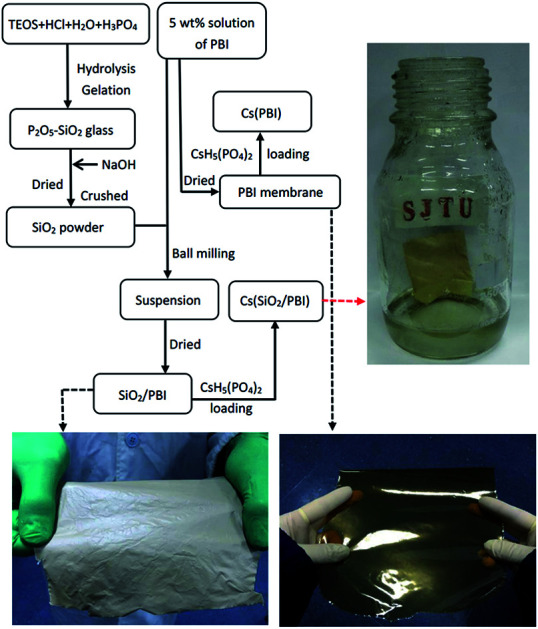

## Introduction

Intermediate-temperature fuel cells (ITFCs), which operate at 120–700 °C, have many excellent features in comparison with low-temperature fuel cells (LTFCs) and high-temperature fuel cells (HTFCs). Compared with LTFCs, ITFCs offer higher tolerance of catalysts to CO, use of lower amount of precious metals, less complicated water management, and electrode kinetics and enhanced catalysis rates.^1–3^ In addition, compared with HTFCs, ITFCs broaden the range of available materials and elongate material lifespans. The heart of a fuel cell is its electrolyte, which determines the operating temperature range of the device. As is well known, among the various fuel cells, the molten carbonate fuel cell (MCFC) is the only one that utilizes a molten salt electrolyte. To maintain the electrolyte in the liquid state, the cell must operate at above 550 °C (the standard working temperature is 650 °C).^4^ Due to the liquid electrolyte of the MCFC, gas seal and low contact resistance are easier to achieve than in other types of fuel cells.^5^ The electrochemical reaction in the MCFC involves the formation of carbonate (CO_3_^2−^) ions at the cathode and their transport to the anode *via* the molten carbonate electrolyte. The molten carbonate electrolyte, typically (Li_0.62_K_0.38_)_2_CO_3_ or (Li_0.52_Na_0.48_)_2_CO_3_, is usually held in a porous ceramic matrix made of lithium aluminate (LiAlO_2_).^6^ Among the various types of H_2_/O_2_ fuel cells, the MCFC has the thickest electrolyte (0.5–1.5 mm).^5^ It is necessary to maintain acceptable mechanical strength, as well as to counteract dissolution of the NiO cathode in molten carbonate, which may result in the formation of Ni dendrites and hence short circuiting between the electrodes.^7^ The thick electrolyte also results in the MCFC showing typical *I*–*V* linear characteristics, with ohmic losses dominating. Therefore, the MCFC offers the low power densities of about 100–150 mW cm^−2^.^8,9^ On the other hand, the high operating temperature of 550–650 °C limits the choice of applicable materials, since the supporting matrices of the molten carbonate electrolyte must be ceramics, which are inherently inflexible and brittle, and thus breakable.

In this work, we have investigated the viability of a molten oxoacid salt as a novel type of fuel cell electrolyte. Pentahydrogen phosphates, MH_5_(PO_4_)_2_ (M = K, Rb, Cs), are a family of oxoacid salts, which are typically comprised of oxyanions linked together by hydrogen bonds.^10,11^ It has been reported that the conductivity of MH_5_(PO_4_)_2_ is independent of the alkaline metal cation at above 160 °C, maintaining an extremely high value of *ca.* 200 mS cm^−1^ due to the molten state.^11^ The oxoacid salt employed in this study, CsH_5_(PO_4_)_2_, melts at around 150 °C, and its melting facilitates anhydrous proton transport.^12^

In comparison with the molten carbonate electrolytes for MCFCs that operate at 500–700 °C, which necessitate the use of a ceramic support matrix (such as γ-LiAlO_2_), the molten proton conductor electrolyte has a lower working temperature range of 150–250 °C. Therefore, we can use a flexible polymer with good mechanical properties as the support matrix for holding the molten CsH_5_(PO_4_)_2_, which can be cast from organic solutions in a convenient and inexpensive process. In this work, polybenzimidazole (PBI) has been utilized as a polymeric matrix due to its superior mechanical strength and chemical stability, and high thermal stability up to 550 °C.^13–15^ The amount of CsH_5_(PO_4_)_2_ proton conductor loaded in the matrices is a key parameter of the electrolyte membranes due to its effect on the proton conductivity, and as much as possible should be loaded to ensure high proton conductivity. Normally, PBI polymer is free from voids, and thus it is difficult to infiltrate a significant amount of CsH_5_(PO_4_)_2_ melt. An efficient technique has been reported for obtaining polymer membranes with a certain distribution of voids, in which doped ceramic particles provide a well-developed porous structure, *i.e.* highly connected voids are formed between the particles, and thus an increase in the amount of ceramic particles results in higher porosity.^13–15^ On the other hand, it has been reported that in composites composed of CsH_5_(PO_4_)_2_ and SiO_2_, the addition of SiO_2_ powder with large surface area leads to the formation of disordered CsH_5_(PO_4_)_2_ at the contact interface and consequently to an increase in proton conductivity by up to 1–2 orders of magnitude compared to that of polycrystalline CsH_5_(PO_4_)_2_.^16,17^ In the present study, a composite matrix has been fabricated by combining PBI polymer and SiO_2_ powder in a mechanical ball-milling process followed by casting from solution, which was then immersed in a CsH_5_(PO_4_)_2_ melt to obtain an electrolyte membrane loaded with CsH_5_(PO_4_)_2_. Results have shown that such electrolyte membranes, in which molten CsH_5_(PO_4_)_2_ is held in a matrix composed of PBI polymer and SiO_2_ powder, display excellent thermal stability, good mechanical properties and high proton conductivity. Furthermore, we demonstrate a molten proton conductor fuel cell (MPCFC) based on the new kind of electrolyte membrane. The MPCFC operating at 200 °C displayed promising electrochemical performances: an open-circuit voltage (OCV) of 1.08 V and a stable output voltage over continuous measurement for 150 h at a constant output current density of 100 mA cm^−2^.

## Results and discussion

### Appearance and morphology

**Fig. 1:**
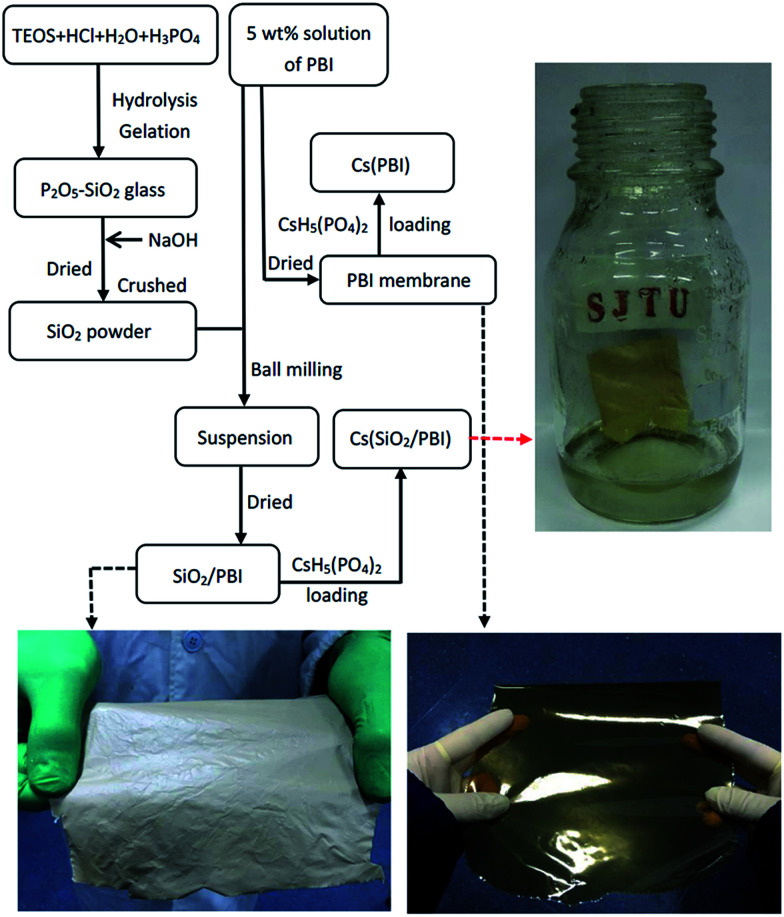
Flow chart of the preparation of all the samples involved in this work. The photographs show pure PBI, an SiO_2_/PBI matrix, and a Cs(SiO_2_/PBI) electrolyte membrane.

Membrane samples were prepared based on the preparation procedure shown in Fig. 1, which features photographs of the materials as insets. As can be seen, the SiO_2_/PBI matrix had a grey, opaque appearance due to the addition of SiO_2_ powder, whereas the pure PBI membrane was dark-brown and transparent. After loading CsH_5_(PO_4_)_2_ within the SiO_2_/PBI matrix, the Cs(SiO_2_/PBI) electrolyte membrane had a light-yellow, opaque appearance. All of the membranes were fully flexible.

**Fig. 2:**
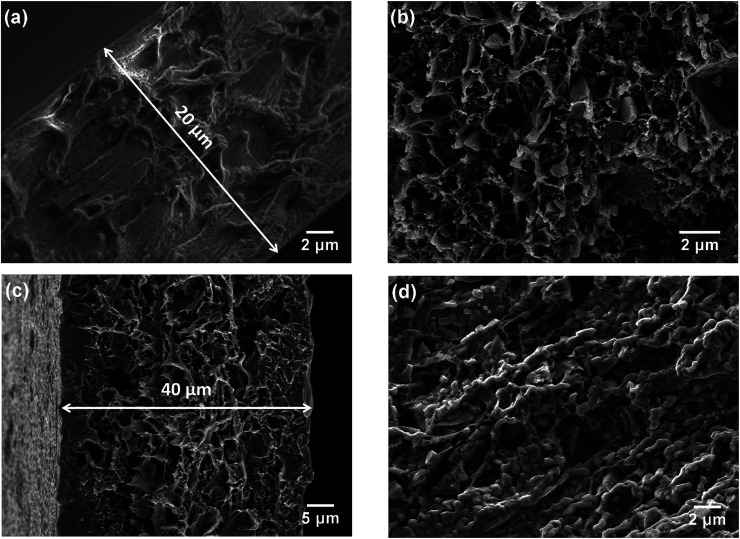
SEM cross-sectional morphologies of (a) the pure PBI membrane, (b) 3SiO_2_/7PBI matrix, (c) as-prepared Cs(3SiO_2_/7PBI) electrolyte membrane, and (d) Cs(3SiO_2_/7PBI) electrolyte membrane after the conductivity test.

The morphologies of the membranes were observed by SEM. In Fig. 2a, it can be seen that the pure PBI membrane was free from visible pores or voids. Fig. 2b is an SEM image of the 3SiO_2_/7PBI matrix membrane, and shows that it consisted of a net-like PBI skeleton and SiO_2_ powder particles with an angular shape smaller than 1 μm embedded therein. Comparison of Fig. 2a and b clearly shows that the addition of SiO_2_ powder promotes the formation of a net-like PBI skeleton with a large number of voids, which will facilitate the infiltration of CsH_5_(PO_4_)_2_ during immersion of the SiO_2_/PBI matrix in a CsH_5_(PO_4_)_2_ melt. The cross-sectional SEM images in Fig. 2a and c reveal that the pure PBI membrane and the Cs(3SiO_2_/7PBI) electrolyte membrane had thicknesses of 20 and 40 μm, respectively. Fig. 2c and d show SEM images of the as-prepared Cs(3SiO_2_/7PBI) electrolyte membrane and after it had been subjected to a conductivity test (from 100 to 260 °C), respectively. The as-prepared Cs (3SiO_2_/7PBI) electrolyte membrane had a cross-sectional structure with voids in the centre and more compaction on either side, which can be rationalized in terms of penetration of the CsH_5_(PO_4_)_2_ melt from both faces towards the centre. Comparing Fig. 2c and d reveals the change in morphology after the conductivity test. At test temperatures up to 260 °C, CsH_5_(PO_4_)_2_ was in its molten liquid state and could flow into the voids in the matrix. This resulted in the formation of a compact electrolyte membrane containing highly connected CsH_5_(PO_4_)_2_, as shown in Fig. 2d. The connected CsH_5_(PO_4_)_2_ provides proton-conducting paths, which are essential for the electrolyte membranes to achieve high proton conductivity. In addition, the infilled molten CsH_5_(PO_4_)_2_ may serve to block gas accessibility within the electrolyte membrane. Such a compact electrolyte membrane can be expected to produce an MPCFC with a high OCV.

### Thermal and mechanical properties

**Fig. 3:**
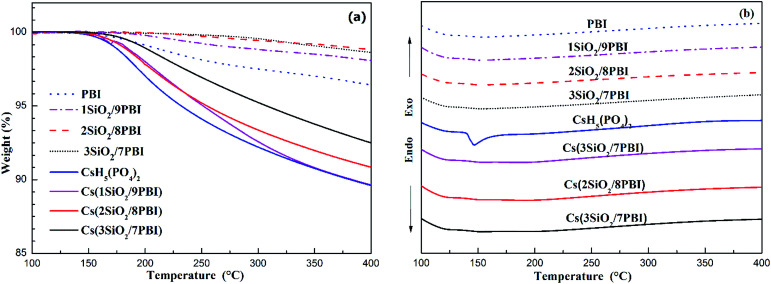
TGA (a) and DSC (b) curves of pure PBI, SiO_2_/PBI, CsH_5_(PO_4_)_2_ and the Cs(SiO_2_/PBI) electrolyte membranes.

The thermal properties of PBI, SiO_2_/PBI, CsH_5_(PO_4_)_2_, and the electrolyte membranes consisting of SiO_2_/PBI matrices loaded with CsH_5_(PO_4_)_2_ were analysed by recording their TGA and DSC curves under a flowing air atmosphere, and the results are given in Fig. 3. As can be seen in the TGA curves in Fig. 3a, the pure PBI membrane showed a weight loss of 3.6% from 120 to 400 °C, which can be attributed to the removal of residual solvents molecules and absorbed water. Compared to the pure PBI membrane, the SiO_2_/PBI matrices showed lower weight losses due to the addition of inorganic SiO_2_ powder. Both the PBI and SiO_2_/PBI membranes showed outstanding thermal stability. As is evident from Fig. 3a, dehydration of CsH_5_(PO_4_)_2_ was initiated after melting under dry conditions,^18^ resulting in a weight loss of 10.5% from 150 to 400 °C. Compared to pure CsH_5_(PO_4_)_2_, the Cs(SiO_2_/PBI) electrolyte membranes showed smaller weight losses, which decreased with increasing content of inorganic SiO_2_ in the matrix. These electrolyte membranes are thus thermally stable and may be used in intermediate-temperature fuel cells.

In the DSC curves in Fig. 3b, CsH_5_(PO_4_)_2_ showed a large endothermic peak at around 150 °C attributable to its melting.^18,19^ In contrast, the Cs(1SiO_2_/9PBI), Cs(2SiO_2_/8PBI), and Cs(3SiO_2_/7PBI) electrolyte membranes loaded with CsH_5_(PO_4_)_2_ showed no corresponding endothermic peaks. According to SEM analyses, it could be speculated that, by immersing SiO_2_/PBI matrices in the CsH_5_(PO_4_)_2_ melt, the molten CsH_5_(PO_4_)_2_ penetrated into the voids within the net-like PBI skeleton, resulting in a highly dispersed distribution thereof. The highly dispersed CsH_5_(PO_4_)_2_ and its disorder at its interface with SiO_2_ particles should be responsible for the disappearance of the endothermic peak corresponding to its melting point.^20^

**Fig. 4:**
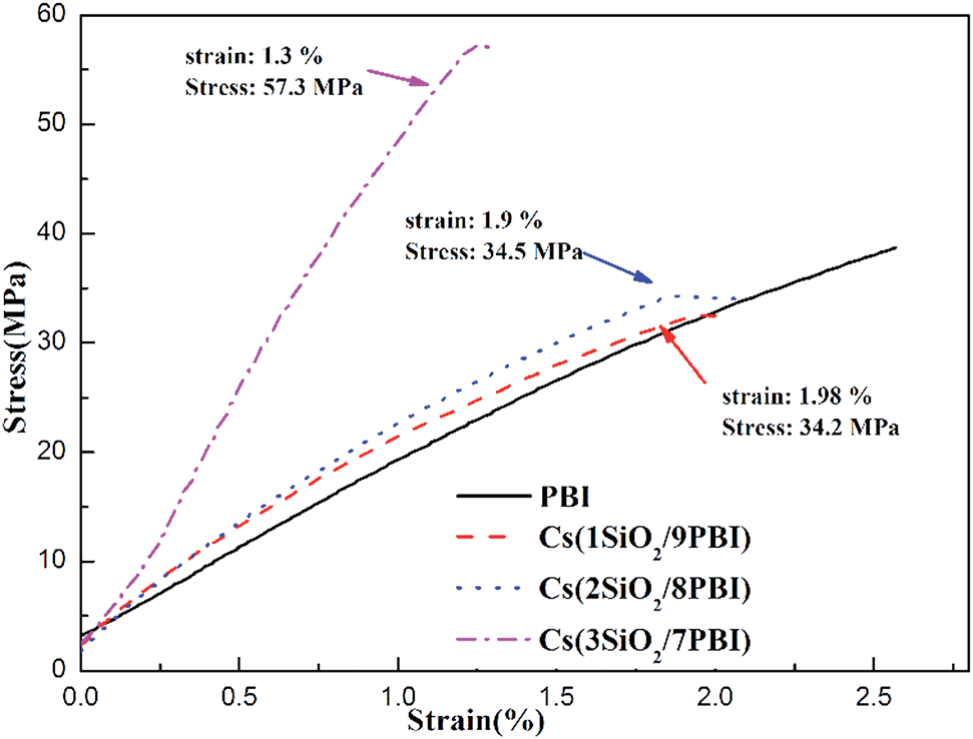
Stress–strain curves of pure PBI and Cs(SiO_2_/PBI) electrolyte membranes.

Good mechanical properties of the electrolyte membrane are essential to meet the needs of MEA fabrication in fuel cells. Fig. 4 shows stress–strain curves of the pure PBI and Cs(SiO_2_/PBI) electrolyte membranes measured at room temperature. It can be seen that all of the membranes had a greater than 34 MPa and an elongation of more than 1.3%, indicating good mechanical properties. It is noteworthy that, due to the high contents of the inorganic components of SiO_2_ powder and CsH_5_(PO_4_)_2_, the Cs(3SiO_2_/7PBI) membrane had a maximum tensile strength of 57.3 MPa, much higher than those of the other membranes.

### Proton conductivities of the electrolyte membranes

**Fig. 5:**
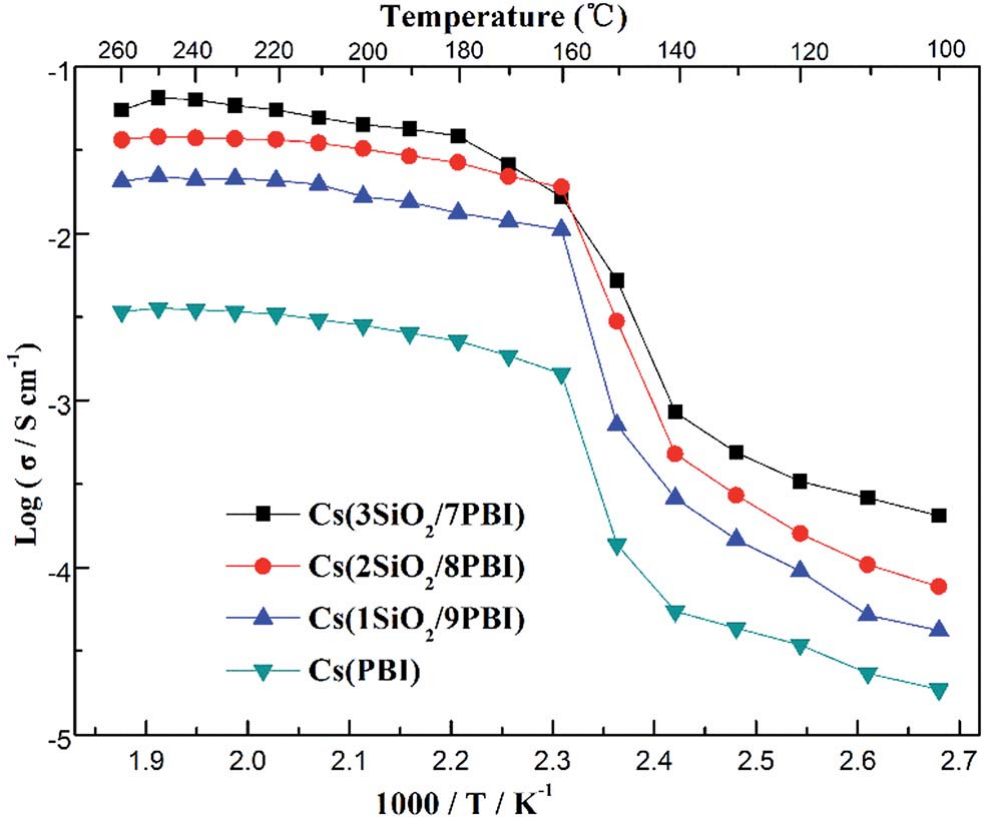
Proton conductivities of the Cs(PBI), Cs(1SiO_2_/9PBI), Cs(2SiO_2_/8PBI), and Cs(3SiO_2_/7PBI) electrolyte membranes under 47% H_2_O/N_2_ atmosphere.

Fig. 5 shows the temperature dependences of the conductivities of various electrolyte membranes consisting of pure PBI and SiO_2_/PBI matrices loaded with CsH_5_(PO_4_)_2_ under 47% H_2_O/N_2_ atmosphere. It can be seen that, for each of the electrolyte membranes, the proton conductivity increased with increasing temperature. More precisely, the temperature changes of the conductivity could be divided into four regions in the studied range between 100 and 260 °C: a gradual increase in the low-temperature range of 100–140 °C, a rapid increase in the region 140–160 °C, a slow increase in the high-temperature range up to 250 °C, and a slight decrease at temperatures over 250 °C. CsH_5_(PO_4_)_2_, as a proton conductor, is responsible for the proton conduction of the electrolyte membranes. The melting point of CsH_5_(PO_4_)_2_ is around 150 °C, as confirmed by the DSC analysis shown in Fig. 3b. In the low-temperature range of 100–140 °C, below its melting point, CsH_5_(PO_4_)_2_ is in the solid state and has a relatively low proton conductivity of 10^−5^–10^−3^ S cm^−1^.^21^ In the region 140–160 °C near its melting point, interfacial CsH_5_(PO_4_)_2_ in contact with the SiO_2_ powder particles and bulk CsH_5_(PO_4_)_2_ successively liquefied due to their different melting points, with that of the interfacial CsH_5_(PO_4_)_2_ being lower than that of the bulk CsH_5_(PO_4_)_2_.^22^ Molten CsH_5_(PO_4_)_2_ is primarily responsible for high proton conduction, resulting in a gradually increasing proton conductivity in the high-temperature range from 160 °C up to 250 °C.^18,23^ Above 250 °C, the decrease in conductivity of the electrolyte membranes may be ascribed to dehydration of the CsH_5_(PO_4_)_2_, which results in a reduction in the carrier concentration. It is noteworthy that the proton conductivities of the Cs(SiO_2_/PBI) electrolyte membranes increased with the increasing content of SiO_2_ powder, and were much higher than that of the Cs(PBI) electrolyte membrane at every temperature investigated. The SiO_2_ powder used had a particle size of <1 μm, as shown in Fig. 2b. From the nitrogen adsorption–desorption isotherm, the specific surface area and pore volume of SiO_2_ powder were evaluated as 343 m^2^ g^−1^ and 0.54 mL g^−1^ respectively. The pore size distribution of the SiO_2_ powder, determined using the BJH method, showed a peak pore size of 6 nm. The addition of SiO_2_ powder to the PBI polymer is seemingly helpful for obtaining electrolyte membranes with high proton conductivities in two respects. The first is the creation of voids within the matrix by the packed particles, as confirmed by SEM observation in Fig. 2b, which allows for greater infusion of the molten CsH_5_(PO_4_)_2_ proton conductor into the SiO_2_/PBI matrix. Indeed, the loading amount increased with increasing content of SiO_2_ powder in the electrolyte membranes, as shown in Table 1, resulting in a higher conductivity. The second factor is the formation of an extensive contact interface between the CsH_5_(PO_4_)_2_ and SiO_2_ powder, with a large specific surface area and an accessible mesoporous structure, such that it could be supposed that disordered CsH_5_(PO_4_)_2_ formed at the interface through interfacial interaction, resulting in an improved conductivity.^22^ Among all of the prepared electrolyte membranes, Cs(3SiO_2_/7PBI) with the highest SiO_2_ content, in which the most CsH_5_(PO_4_)_2_ was loaded, showed the highest conductivity of 64.8 mS cm^−1^ at 250 °C.

**Table 1:** Designations, compositions, and matrices of the electrolyte membranes loaded with CsH_5_(PO_4_)_2_

Electrolyte membrane	Matrix	SiO_2_ content (wt%)	CsH_5_(PO_4_)_2_ loading level (wt%)
Cs(PBI)	Pure PBI	0	103
Cs(1SiO_2_/9PBI)	1SiO_2_/9PBI	10	241
Cs(2SiO_2_/8PBI	2SiO_2_/8PBI	20	373
Cs(3SiO_2_/7PBI)	3SiO_2_/7PBI	30	468

#### Characterization of an MPAFC

**Fig. 6:**
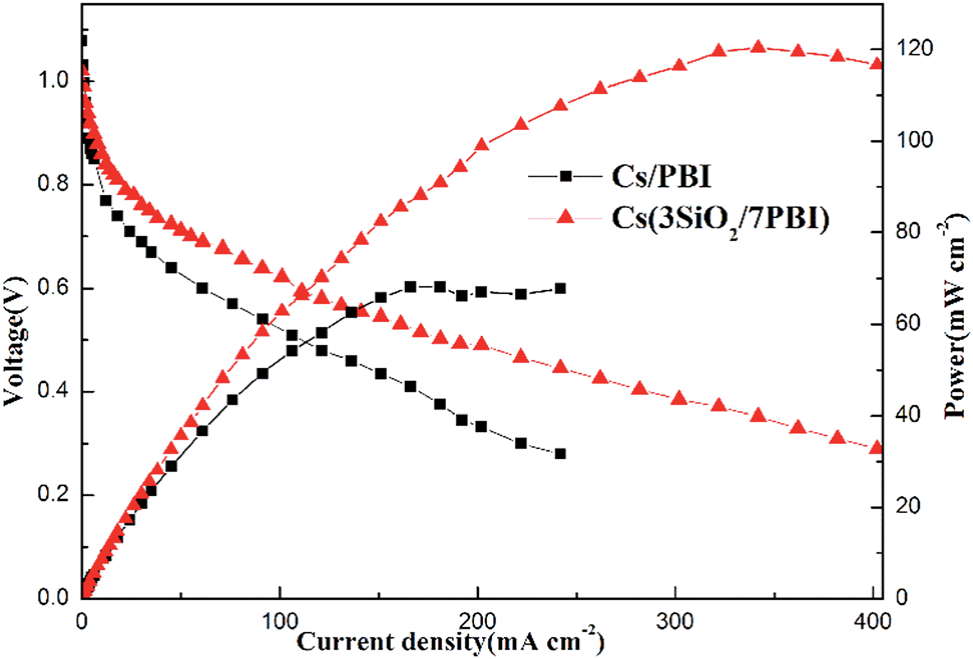
Current–voltage (*I*–*V*) and current–power (*I*–*P*) curves of single cells assembled with the Cs(PBI) and Cs(3SiO_2_/7PBI) electrolyte membranes, which were operated at 200 °C. The gas flow rates of H_2_ and O_2_ were set at 60 cm^3^ min^−1^ at 1 bar. Humidification of H_2_ was achieved by feeding water at a flow rate of 0.12 mL min^−1^ into the gas flow.

Considering its superior thermal, mechanical, and proton conductivity performances, the Cs(3SiO_2_/7PBI) membrane was selected to assemble a single cell of an MPAFC. For comparison, an MPAFC was also prepared using a Cs(PBI) membrane. Both MPAFCs were tested by feeding humidified H_2_ to the anode and dry O_2_ to the cathode at 200 °C, a temperature above the melting point of CsH_5_(PO_4_)_2_. Polarization and power output curves were recorded to evaluate the overall performance of the fuel cells, as shown in Fig. 6. The OCVs of the cells based on the Cs(3SiO_2_/7PBI) and Cs(PBI) electrolyte membranes were 1.08 V and 1.02 V, respectively, higher than those typically observed for polymer-based fuel cells: 0.9–1.0 V.^24^ It is speculated that, for the electrolyte membranes loaded with CsH_5_(PO_4_)_2_, the CsH_5_(PO_4_)_2_ is in its molten liquid state at the fuel cell operating temperature of 200 °C, thus allowing it to fill the voids in the matrix, as previously analysed by SEM. This blocks gas leakage through the membrane, resulting in the high OCV. The differences between the measured values and the theoretical OCV (about 1.15 V) may be attributed to small leaks across the fuel cell seals.

The fuel cells equipped with Cs(3SiO_2_/7PBI) and Cs(PBI) electrolyte membranes gave peak power densities of 120 and 68 mW cm^−2^, respectively. Clearly, the former is higher than the latter. The main reason is probably that, compared to the Cs(PBI) membrane, the Cs(3SiO_2_/7PBI) membrane with a higher loaded content of CsH_5_(PO_4_)_2_ has a higher proton conductivity under the fuel cell operating conditions, resulting in a lower ohmic resistance.

**Fig. 7:**
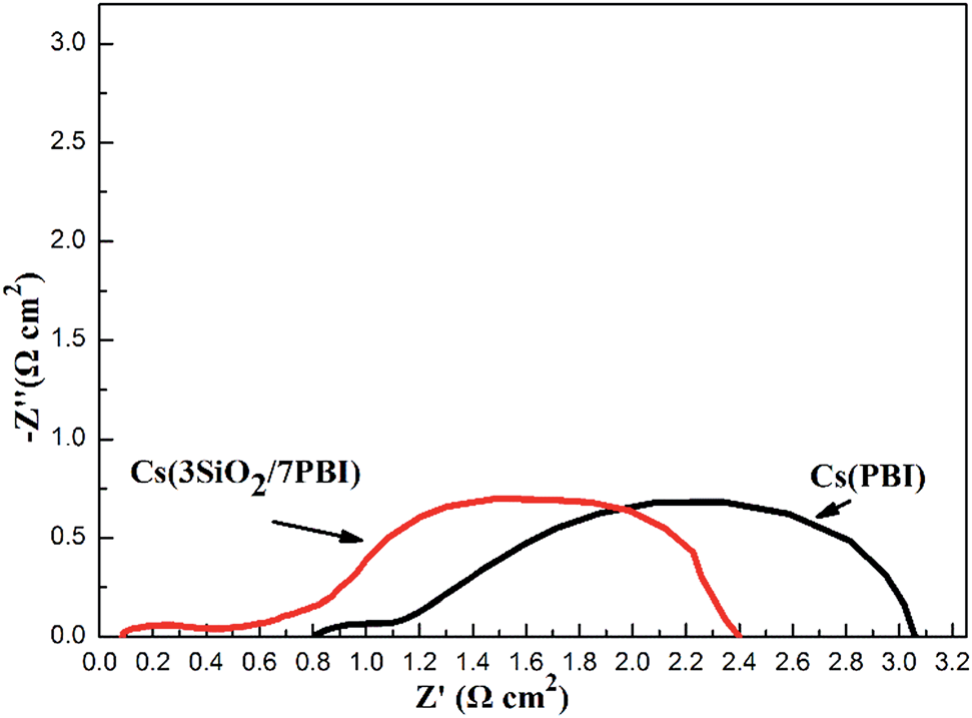
Impedance spectra of MPAFCs with Cs(PBI) and Cs(3SiO_2_/7PBI) measured at 100 mA cm^−2^; the fuel cell was operated at 200 °C under humidified H_2_ and dry O_2_ gases at the anode and cathode, respectively.

An impedance analysis of the MPAFCs was carried out with a current load of 100 mA cm^−2^ and impedance spectra are shown in Fig. 7. The intercept in the high-frequency domain on the real axis represents the ohmic resistance (*R*_Ω_) of the fuel cell, which is mainly ascribed to the membrane, and it is 0.084 Ω cm^2^ for the Cs(3SiO_2_/7PBI) membrane and 0.81 Ω cm^2^ for the Cs(PBI) membrane. Cs(3SiO_2_/7PBI) and Cs(PBI) electrolyte membranes have the thicknesses of 42 and 23 μm, respectively, and thus their proton conductivity could be calculated to be 50 and 2.84 mS cm^−1^, which are in accordance with data showed in Fig. 5. The diameter of the semicircle in the low-frequency domain is the electrode resistance (*R*_e_) from both the anode and cathode, which is 2.32 Ω cm^2^ for the Cs(3SiO_2_/7PBI) membrane and 2.30 Ω cm^2^ for the Cs(PBI) membrane. It is well known that creating the so-called triplephase boundaries, in which the proton conductor, catalysts, and reactants are in close contact with each other, is critical in reducing electrode resistance. In the present study, the used commercial GDEs contain Nafion resin as proton conductor. The fuel cell operating temperature of 200 °C could cause the deterioration of the proton transfer ability, resulting in the increasing electrode resistance. It is believed that the introduction of an intermediate-temperature proton conductor such as CsH_5_(PO_4_)_2_ instead of Nafion® resin within the GDEs should be able to achieve better triplephase boundaries, leading to the reduction of electrode resistance.

**Fig. 8:**
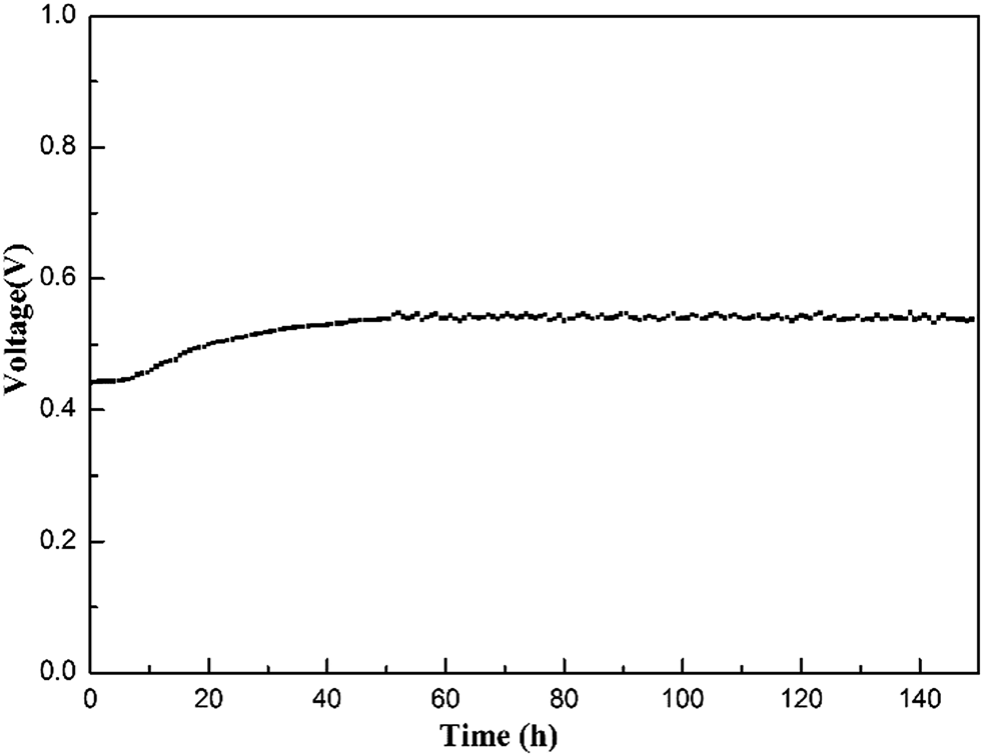
Output voltage of an MPAFC with a Cs(3SiO_2_/7PBI) electrolyte membrane as a function of time for 150 h at an output current density of 100 mA cm^−2^. The fuel cell was operated at 200 °C under humidified H_2_ and dry O_2_ gases at the anode and cathode, respectively.

To evaluate the stability of the electrolyte membrane operating in a fuel cell, a longevity experiment was conducted at 200 °C on an MPAFC equipped with the Cs(3SiO_2_/7PBI) electrolyte membrane. Fig. 8 shows the stability of the output voltage with time at a constant output current density of 100 mA cm^−2^. After an initial increase from *ca.* 0.44 to 0.55 V, during the continuous measurement up to 150 h, the fuel cell showed good stability at around 0.55 V, indicating high stability of the electrolyte membrane under the operating conditions.

## Conclusions

In the present study, we have succeeded in preparing proton-conducting electrolyte membranes loaded with molten CsH_5_(PO_4_)_2_, and have constructed a working fuel cell. Although further improvements, such as better MEA design, are definitely required, the presented results proved that molten proton conductor electrolyte membranes show advantages of non-permeability of fuel gases, sufficient proton conductivity, high thermal stability, good mechanical properties, and low fabrication cost.

So far, various ITFCs including MCFC and phosphoric acid fuel cell (PAFC) have been developed, and they have varying operating temperatures, covering the intermediate-temperature range from 120 °C up to 700 °C. Table 2 summarizes the operating temperature, electrolyte, matrix and mobile ion information of the MPCFC using the molten proton conductor electrolyte in comparison with the MCFC and PAFC as conventional ITFCs. Under the high operation temperatures of 500–700 °C, molten carbonate fuel cell suffers from a high stresses due to thermal cycling. On the other hand, the advantages of using the PBI polymeric matrix to retain the electrolyte in liquid form in MPCFC and PAFC over the ceramic matrix such as the LiAlO_2_ in MCFC and the SiC matrix in PAFC include, easy handling, thinner electrolyte thickness, less cost and better tolerance towards pressure differences between cathode and anode.

**Table 2:** ITFCs types and selected features

Type	Operating temperature	Electrolyte	Matrix	Mobile ion
MPCFC	150–250 °C	Molten proton conductor (CsH_5_(PO_4_)_2_)	PBI (polymer)	H^+^
MCFC	500–700 °C	Molten carbonate ((Na, K)_2_CO_3_)	LiAlO_2_ (ceramic)	CO_3_^2−^
PAFC	120–200 °C	H_3_PO_4_	SiC (ceramic)	H^+^
	120–200 °C	H_3_PO_4_	PBI (polymer)	H^+^

One of the main drawbacks of fuel cells that are hindering their widespread commercialization are hydrogen infrastructure. Methanol is such a liquid hydrogen carrier, and has the lowest reforming temperature, around 200–300 °C compared to other hydrocarbons. A way of using methanol is by means of methanol reforming, where it is used to produce hydrogen rich mixture of gases that can be utilized in fuel cells. Due to the mismatch on the operating temperature between the low-temperature methanol reformer (200–300 °C) and the PAFC (120–200 °C), the heat released by the PAFC is mostly wasted, and thus the simplest and most efficient way to achieve heat integration is having both ITFCs and methanol reformer operated at same temperature.^25^ The MPCFC using molten proton conductor electrolyte can operate at a temperature from 150 °C up to 250 °C, which allows matching methanol reforming (200–300 °C), making it very attractive for the possibility of its integration with methanol reformer. It could be expected that new types of MPCFCs using the molten proton conductor electrolytes capable of operating at intermediate temperatures would considerably affect the direction of current intermediate-temperature fuel cell research.

## Experimental section

### Materials

Si(OC_2_H_5_)_4_ (TEOS), NaOH, HCl, H_3_PO_4_ (85 wt% in water), LiCl, and Cs_2_CO_3_ (99.9% trace metals basis) were purchased from Shanghai Sinopharm Chemical Reagent Co., Ltd. *N*,*N*-dimethyl-acetamide (DMAc) was purchased from Shanghai Lingfeng Chemical Reagent Co. Ltd. Polybenzimidazole (PBI) was purchased from Shanghai Shengjun Polymer Technologies Co. Ltd. Silver paste (DAD-40) was purchased from the Shanghai Research Institute of Synthetic Resins. All materials were used as received without further purification.

### Preparation

#### Synthesis of SiO_2_ powder

SiO_2_ powder with a mesoporous structure was prepared by a sol–gel route employing phosphoric acid as a pore-forming template, as reported elsewhere.^17,26^ Briefly, a mixture of TEOS, deionized water, and hydrochloric acid in a molar ratio of 1 : 4 : 4 × 10^−3^ (TEOS : H_2_O : HCl) was firstly prepared and stirred for 30 min at room temperature, and then H_3_PO_4_ was added to give a molar ratio of 7 : 3 (TEOS : H_3_PO_4_) and the mixture was stirred for 1 h. The obtained transparent solution was then transferred to a Teflon vessel and left to stand at room temperature until gelation occurred. Neutralization of the acidic gel was a necessary step to avoid the precipitation of PBI with a basic amino group (–NH_2_) when the acidic inorganic material was added to the solution. The SiO_2_ gel monolith containing phosphoric acid was immersed in 2 mol L^−1^ NaOH solution to neutralize the H_3_PO_4_ contained therein, then washed repeatedly with distilled water until the washings reached neutral pH, and subsequently dried at 120 °C for 24 h, so that a neutral SiO_2_ gel monolith was obtained. Finally, the SiO_2_ gel monolith was crushed to SiO_2_ powder using an agate mortar, and this powder was kept under a dry atmosphere before use.

#### Synthesis of CsH_5_(PO_4_)_2_

CsH_5_(PO_4_)_2_ was synthesized from Cs_2_CO_3_ and H_3_PO_4_ as the starting materials. An aqueous solution of Cs_2_CO_3_/H_3_PO_4_ in a molar ratio of 1 : 4 was concentrated to dryness overnight at 100 °C and then the resultant white solid was kept under a dry atmosphere prior to use.

#### Preparation of pure PBI and SiO_2_/PBI matrix membranes

PBI powder and LiCl as a stabilizer (PBI : LiCl, 100 : 3, w/w) were dissolved in DMAc with vigorous stirring at 120 °C for 5 h to form a 5 wt% solution. This solution was then cast onto a glass plate. A pure PBI membrane was obtained by initially drying at 70 °C for 10 h, then at 120 °C for 10 h, and finally at 120 °C under vacuum overnight. It was then peeled off from the glass plate, and washed in boiling water for 10 h to remove LiCl and residual DMAc.

SiO_2_/PBI matrix membranes were prepared according to the following procedure. First, the requisite amounts of SiO_2_ were incorporated into 5 wt% PBI solutions. The obtained mixtures of PBI and SiO_2_ were milled in a planetary ball-milling apparatus (QM-3SP2, NanDa Instrument Plant) for 24 h at 580 rpm and then cast onto glass plates. The SiO_2_/PBI composite matrices were obtained by initially drying at 70 °C for 10 h, then at 120 °C for 10 h, and finally at 120 °C under vacuum overnight. They were then peeled off from the glass plate, and washed in boiling water for 10 h to remove LiCl and residual DMAc.

To indicate the different weight ratios of SiO_2_ powder and PBI polymer in the SiO_2_/PBI matrices, such as 1 : 9, 2 : 8, and 3 : 7, they were designated as 1SiO_2_/9PBI, 2SiO_2_/8PBI, and 3SiO_2_/7PBI, respectively.

#### Preparation of SiO_2_/PBI matrix and pure PBI electrolyte membranes loaded with CsH_5_(PO_4_)_2_

The SiO_2_/PBI matrix membranes were immersed in a CsH_5_(PO_4_)_2_ melt for 48 h at 160 °C for loading with CsH_5_(PO_4_)_2_, and the obtained electrolyte membranes were designated as Cs(SiO_2_/PBI). The CsH_5_(PO_4_)_2_ loading level was defined as the weight change before and after immersing in the melt and was calculated using the following equation: *W* (%) = (*m* − *m*_0_)/*m*_0_ × 100 (%), where *m*_0_ and *m* are weights of samples without and with CsH_5_(PO_4_)_2_, respectively. For comparison, an electrolyte membrane consisting of pure PBI loaded with CsH_5_(PO_4_)_2_ was prepared in the same manner, and designated as Cs(PBI). A flow chart of the preparation of the electrolyte membranes is shown in Fig. 1. The designations, compositions, and matrices of all of the electrolyte membranes are listed in Table 1.

#### Characterization

TGA was performed on a simultaneous DSC-TGA apparatus (TA 157Q600). All samples were pre-heated to 120 °C, cooled to 90 °C, and then heated to 400 °C at a rate of 10 °C min^−1^ in a flow of air of 100 mL min^−1^. The mechanical properties of the electrolyte membranes were assessed on a Perkin-Elmer dynamic mechanical analyser (DMA 8000) using a DMA-controlled force module with a stretching speed of 0.3 N min^−1^ at room temperature. Membrane samples of a size 25 × 5 mm^2^ were used for the tests. The cross-sectional morphologies of the membrane samples were analysed by means of a scanning electron microscope (SEM, JSM-7800F). The method of liquid nitrogen brittle fracture was used to acquire cross-sections of the membranes. The average pore size, pore volume, and specific surface area of the SiO_2_ powder were evaluated by measuring N_2_ adsorption–desorption isotherms with a Micromeritics ASAP2460 apparatus.

The proton conductivities of the electrolyte membranes were determined by means of an impedance/gain phase analyser (SI 1260, Solartron) over the frequency range from 10^−1^ to 10^7^ Hz with a voltage amplitude of 20 mV. Measurements were carried out over the temperature range from 100 to 260 °C under a 47% H_2_O/N_2_ atmosphere generated by bubbling dry N_2_ through water at 80 °C. A two-electrode a.c. method described in a previous study^27^ was applied for these measurements. Two parallel silver stripe electrodes connected with gold wires were obtained by painting silver paste on each electrolyte membrane. To obtain a steady state, a time interval of 30 min between adjacent temperatures (testing temperature elevated by 10 °C) was selected. The electrolyte resistance was estimated from the intercept on the real axis at the high-frequency end of the impedance spectra. The proton conductivities of the electrolyte membranes were calculated according to the following equation:

1 *σ* = *a*/*dLR*

where *σ* is the conductivity, *a* is the distance between the two parallel electrodes, *L* is the length of the silver stripe electrodes, *d* is the thickness of the electrolyte membrane, and *R* is the measured resistance of the electrolyte membrane.

Single cells of the MPCFC were fabricated using the Cs(PBI) and Cs(3SiO_2_/7PBI) electrolyte membranes. The electrolyte membranes were sandwiched between two gas diffusion electrodes (GDEs) with Pt loadings of 0.5 mg cm^−2^ (S10CC, SGL Group) without a hot-pressing procedure to form a membrane-electrode assembly (MEA) with an active area of 5 cm^2^. These single cells were fueled with humidified hydrogen at the anode and dry oxygen at the cathode, respectively. The gas flow rates of H_2_ and O_2_ were both set at 60 cm^3^ min^−1^ at 1 bar. Humidification of H_2_ at the anode was achieved by feeding water at a flow rate of 0.12 ml min^−1^ into the gas flow. The MPCFCs were characterized by polarization curves and electrochemical impedance spectroscopy (EIS). EIS were obtained at 100 mA cm^−2^, between 10^−1^ and 10^5^ Hz with a perturbation amplitude of 5 mV. A durability test of the MPCFC assembled with the Cs(3SiO_2_/7PBI) electrolyte membrane was conducted by operating under a constant output current density of 100 mA cm^−2^ at 200 °C.

## Conflicts of interest

There are no conflicts to declare.

## Supplementary Material
