# Correction: A proton conductor electrolyte based on molten CsH_5_(PO_4_)_2_ for intermediate-temperature fuel cells

**DOI:** 10.1039/c8ra90015a

**Published:** 2018-02-20

**Authors:** Xiaojing Chen, Yichong Zhang, Paulo Ribeirinha, Haibin Li, Xiangyang Kong, Marta Boaventura

**Affiliations:** School of Materials Science and Engineering, Shanghai Jiao Tong University 800 Dong Chuan Road Shanghai 200240 China; State Key Laboratory of Ocean Engineering, Shanghai Jiao Tong University 800 Dongchuan Road Shanghai 200240 China haibinli@sjtu.edu.cn; Collaborative Innovation Center for Advanced Ship and Deep-Sea Exploration, Shanghai Jiao Tong University 800 Dongchuan Road Shanghai 200240 China; LEPABE, Faculdade de Engenharia, Universidade do Porto Rua Dr. Roberto Frias 4200-465 Porto Portugal marta.boaventura@fe.up.pt

## Abstract

Correction for ‘A proton conductor electrolyte based on molten CsH_5_(PO_4_)_2_ for intermediate-temperature fuel cells’ by Xiaojing Chen *et al.*, *RSC Adv.*, 2018, **8**, 5225–5232.

The authors regret that Paulo Ribeirinha’s name was spelled incorrectly in the original article. The correct spelling of all author names is presented above.

The Royal Society of Chemistry apologises for these errors and any consequent inconvenience to authors and readers.

## Supplementary Material

